# Progesterone-Based Therapy Protects Against Influenza by Promoting Lung Repair and Recovery in Females

**DOI:** 10.1371/journal.ppat.1005840

**Published:** 2016-09-15

**Authors:** Olivia J. Hall, Nathachit Limjunyawong, Meghan S. Vermillion, Dionne P. Robinson, Nicholas Wohlgemuth, Andrew Pekosz, Wayne Mitzner, Sabra L. Klein

**Affiliations:** 1 W. Harry Feinstone Department of Molecular Microbiology and Immunology, The Johns Hopkins Bloomberg School of Public Health, Baltimore, Maryland, United States of America; 2 Department of Environmental Health Sciences, The Johns Hopkins Bloomberg School of Public Health, Baltimore, Maryland, United States of America; 3 Department of Molecular and Comparative Pathobiology, The Johns Hopkins University School of Medicine, Baltimore, Maryland, United States of America; 4 Department of Biochemistry and Molecular Biology, The Johns Hopkins Bloomberg School of Public Health, Baltimore, Maryland, United States of America; St. Jude Children’s Research Hospital, UNITED STATES

## Abstract

Over 100 million women use progesterone therapies worldwide. Despite having immunomodulatory and repair properties, their effects on the outcome of viral diseases outside of the reproductive tract have not been evaluated. Administration of exogenous progesterone (at concentrations that mimic the luteal phase) to progesterone-depleted adult female mice conferred protection from both lethal and sublethal influenza A virus (IAV) infection. Progesterone treatment altered the inflammatory environment of the lungs, but had no effects on viral load. Progesterone treatment promoted faster recovery by increasing TGF-β, IL-6, IL-22, numbers of regulatory Th17 cells expressing CD39, and cellular proliferation, reducing protein leakage into the airway, improving pulmonary function, and upregulating the epidermal growth factor amphiregulin (AREG) in the lungs. Administration of rAREG to progesterone-depleted females promoted pulmonary repair and improved the outcome of IAV infection. Progesterone-treatment of AREG-deficient females could not restore protection, indicating that progesterone-mediated induction of AREG caused repair in the lungs and accelerated recovery from IAV infection. Repair and production of AREG by damaged respiratory epithelial cell cultures *in vitro* was increased by progesterone. Our results illustrate that progesterone is a critical host factor mediating production of AREG by epithelial cells and pulmonary tissue repair following infection, which has important implications for women’s health.

## Author Summary

Worldwide, the use of hormonal contraceptives is on the rise as a primary intervention for improving women’s health outcomes through reduced maternal mortality and increased childhood survival. There are many hormone contraceptive formulations, all of which contain some form of progesterone. Although the effects of hormone contraceptives and progesterone, specifically, have been evaluated in the context of infections of the reproductive tract, the effects of progesterone at other mucosal sites, including the respiratory tract have not been systematically evaluated. We have made the novel observation that administration of progesterone to female mice depleted of progesterone confers protection against both lethal and sublethal influenza A virus infection. In particular, progesterone reduces pulmonary inflammation, improves lung function, repairs the damaged lung epithelium, and promotes faster recovery following influenza A virus infection. Progesterone causes protection against severe outcome from influenza by inducing production of the epidermal growth factor, amphiregulin, by respiratory epithelial cells. This study provides insight into a novel mechanistic role of progesterone in the lungs and illustrates that sex hormone exposure, including through the use of hormonal contraceptives, has significant health effects beyond the reproductive tract.

## Introduction

Hormonal contraceptives are listed as an essential medication by the World Health Organization (WHO)[1] because of the profound benefits these compounds can have on women’s health outcomes, including decreased rates of maternal mortality and improved perinatal outcomes and child survival, by widening the intervals between pregnancies [2]. Hormonal contraceptive formulations vary, but all contain some form of progesterone (P4) either alone or in combination with estrogen. There are currently over 100 million young adult women on P4-based contraceptives worldwide [3], with the WHO projecting that over 800 million women will be using contraceptives, including P4-based contraceptives, by 2030 [2]. Despite the staggering numbers of women taking these compounds, very few studies evaluate the impact of contraceptives on responses to infection or vaccination, especially in non-sexually transmitted diseases.

Natural P4, produced by the ovaries during reproductive cycles, or synthetic P4 analogues found in contraceptives, signal primarily through progesterone receptors present on many cells in the body, including immune cells (e.g., NK cells, macrophages, dendritic cells (DCs), and T cells) as well as non-immune cells, such as epithelial cells, endothelial cells, and neuronal cells [4, 5]. Human, animal, and *in vitro* studies show that P4 can alter the immune environment and promote homeostasis by decreasing inflammation and inducing anti-inflammatory responses. For example, in the presence of P4, macrophages and DCs have a lower state of activation, produce higher levels of anti-inflammatory cytokines, such as IL-10, and produce lower amounts of proinflammatory cytokines, such as IL-1β and TNF-α, as compared with placebo treated cells [6, 7]. When either mice or cord blood cells from humans are treated with P4, the percentages of Foxp3+ regulatory T cells (Tregs) increase [8, 9]. Although the immunomodulatory effects of P4-based therapies in the form of contraception have been studied in the context of sexually transmitted infections, including HIV and herpes simplex virus [10–12], the impact of P4 on the outcome of viral infectious diseases outside of the reproductive tract has not been considered in either humans or animal models.

Influenza A viruses (IAVs) primarily infect respiratory epithelial cells and induce the production of proinflammatory cytokines and chemokines that recruit immune cells, causing a local proinflammatory environment [13]. Infiltration and activation of CD4+ and CD8+ T cells, while necessary for the clearance of IAVs [13–15], can trigger inflammation and lead to tissue damage and severe outcomes from IAV infection [16]. Protection requires a balance between inflammatory responses generated to control virus replication and eliminate virus-infected cells with responses that mediate the repair of damaged areas of the lung. Repair involves a complex interplay among many cell types, cytokines, chemokines, growth factors, and extracellular matrix proteins that remodel tissue after acute injury, such as IAV infection [17]. Amphiregulin (AREG) is an epidermal growth factor that has emerged as a significant mediator of tissue repair at mucosal sites, including the lungs [18, 19], gastrointestinal tract [20, 21], and reproductive tract [22, 23]. Many immune cells produce AREG, but epithelial cells are the principle producer of AREG following inflammation or tissue injury [24]. If P4 can downregulate inflammatory immune responses and promote regulatory or tissue repair responses, then this hormone, at concentrations that reflect the luteal phase of the reproductive cycle, may improve the outcome of IAV infection.

Epidemiological and experimental evidence suggest that young adult females suffer a worse outcome than males following IAV infection, which in mice is associated with infection-induced suppression of reproductive hormones and excessive inflammatory immune responses in females [25–27]. In addition to influenza, young adult females suffer a worse outcome than males from several autoimmune diseases, including multiple sclerosis [28]. Paradoxically, a growing body of literature reveals that exogenous treatment of females (both humans and mice) with either estrogens or P4 limits inflammation and protects against infectious and autoimmune diseases by decreasing inflammation and promoting repair [25, 29–31]. In this series of studies, we show that treatment with sustained physiological doses of P4 protects females against IAV by reducing inflammation and improving pulmonary function, primarily through upregulation of AREG in epithelial cells. The observation that P4 regulates the cellular and molecular mediators of tissue repair at a mucosal site outside of the reproductive tract to restore tissue homeostasis after infection or injury has broad implications for women’s health.

## Results

### Progesterone limits lung pathology and protects female mice against lethal IAV infection

**Fig 1:**
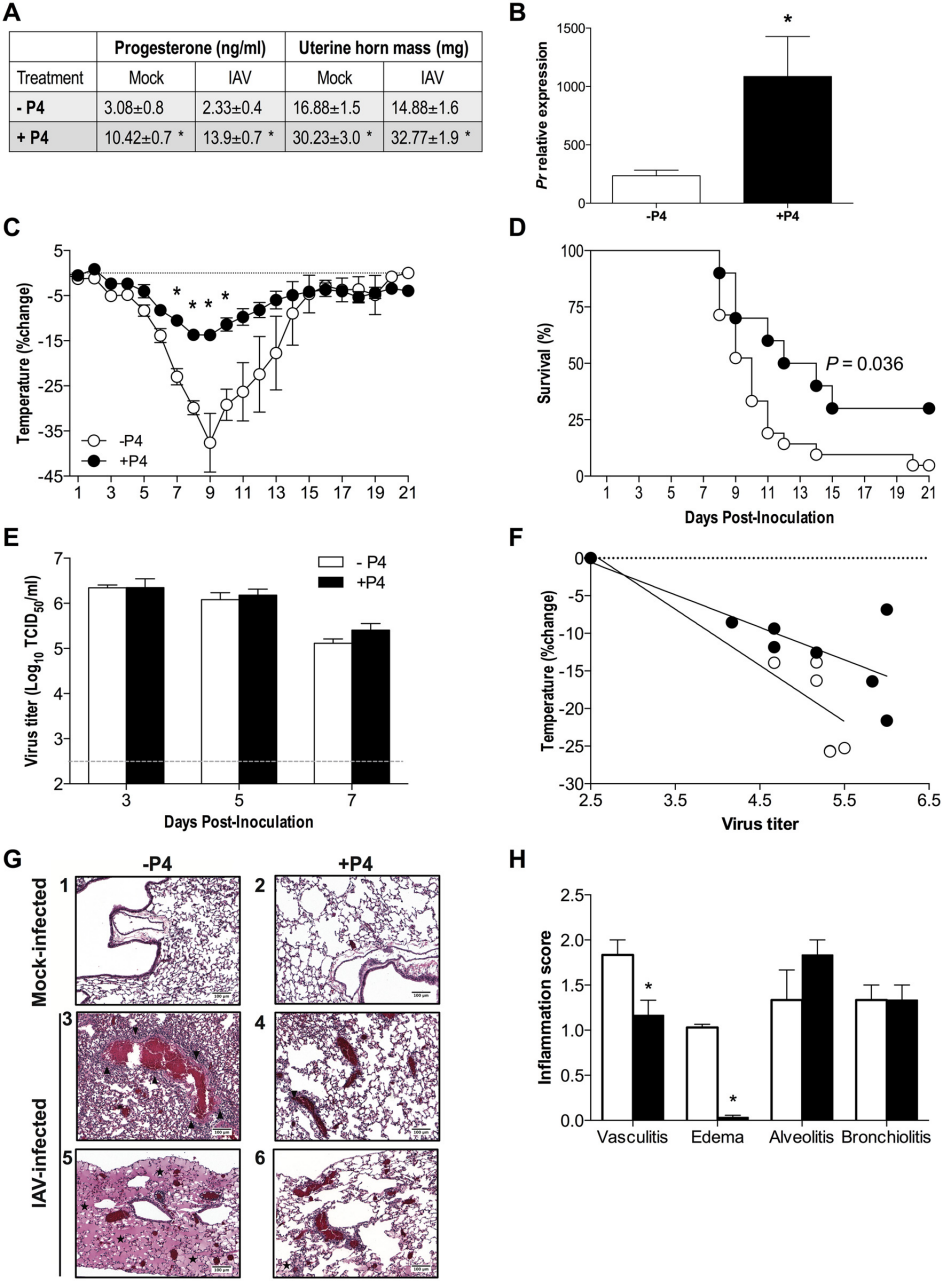
Progesterone (P4) protects adult female mice against lethal IAV infection. Adult female mice were ovariectomized, treated with placebo (-P4) or exogenous P4 (+P4), and inoculated with lethal IAV or mock-infected. Serum was collected at 3, 5, 7, and 21 days post-inoculation (dpi) and P4 concentrations (mock n = 5, IAV n = 20–22 [i.e., n = 5–7 per dpi]) were analyzed by radioimmunoassay, and uterine horns (mock n = 13, IAV = 35–38 i.e., n = 12–14 dpi time-point) were weighed (A). Lungs were harvested at days 3, 5, or 7 dpi and mRNA expression of the progesterone receptor (*Pr*) was measured and normalized to GAPDH and mock-infected animals using the ΔΔCt method (B). Values for each measure (A and B) did not differ between dpi and are shown as aggregates. Mice (-P4 n = 20, +P4 n = 10) were monitored daily for changes in rectal body temperature (C) and survival (D) for 21 dpi. Infectious virus titers in the lungs were measured at 3, 5, or 7 dpi (E; n = 8-10/treatment/dpi). The correlation between changes in body temperature and virus titers at 7dpi, as a measure of disease tolerance, was quantified using a linear regression model (F; n = 12/treatment). H&E stained lung sections collected at 7dpi from mock-infected (G, panel 1 and 2) and IAV-infected females (G, panels 3–6) were scored for inflammation. Alveolitis (G panel 3 and 4, indicated by black triangles) and edema are shown (G panels 5 and 6, indicated by black stars), as well as corresponding histopathogical scores on a scale from 0–3 (H, n = 3/treatment, 10 fields/animal, 10X magnification). Data represent means ± SEM from two independent experiments and significant differences are represented by asterisks (*).

To analyze the effects of P4 on morbidity and mortality in female mice, we depleted P4 by removing the ovaries and replaced P4 with subcutaneous pellets that delivered a continuous dose of physiological levels of P4 over the course of 21 days. Mice were subsequently mock-infected or infected with a dose of IAV (PR8) that is uniformly lethal for P4-depleted mice. Circulating levels of P4 and uterine horn mass, a biomarker of circulating P4 levels [32], were assessed over the course of infection to confirm the continuous effects of hormone replacement. Exogenous replacement of P4 significantly increased and sustained plasma P4 concentrations within the normal physiological range [33] throughout the duration of the study. Both mock- and IAV-infected females treated with exogenous P4 had higher circulating concentrations of P4, greater uterine horn mass, and higher expression of progesterone receptors (*Prs*) in the lungs than either mock or IAV-infected females treated with placebo throughout the 21 days (Fig 1A and 1B; *P*<0.05).

During the course of IAV infection, treatment of female mice with P4 mitigated the effects of infection on morbidity and mortality (Fig 1C and 1D; *P*<0.05), with the average day of death being later for females treated with P4 (11.14±1.0 days post-infection [dpi]) as compared to placebo-treated females (9.5±0.6 dpi) (*P*<0.05). Progesterone treatment did not alter virus titers over the course of the first week of infection as compared to placebo treatment (Fig 1E), suggesting that P4 did not render females more resistant to IAV infection. To test whether P4 improved survival during IAV infection by making females more tolerant to the negative consequences of infection on host health, we analyzed the interaction between virus titers and body temperature during peak disease (7dpi) [34]. Females treated with P4 suffered less hypothermia relative to their pulmonary viral load than the placebo-treated females, suggesting that P4 made females more tolerant of IAV infection (Fig 1F; *P*<0.05). To test the hypothesis that P4 may increase tolerance by reducing inflammation and damage in the lung, pulmonary tissue was evaluated for vasculitis, bronchiolitis, alveolitis, and edema. In mock-infected animals, P4 alone did not result in changes in any of the parameters examined (Fig 1G [panels 1 and 2]). Seven days post-infection with IAV, treatment with P4 decreased vasculitis (Fig 1G [panels 3 and 4] and 1H) and edema (Fig 1G [panels 5 and 6] and 1H) as compared to the placebo-treated mice (*P*<0.05). Progesterone improved the outcome of lethal IAV infection by limiting lung inflammation and damage, but not virus replication.

Virus-specific CD8+ T cells are necessary for clearance of IAV but can also contribute to immunopathology [35, 36]. Although the total numbers of CD8+ T cells increased in all females following IAV infection, the total number of CD8+ T cells, the number of IAV-specific CD8+ T cells, and the production of IFN-γ and TNF-α by virus-specific CD8+ T cells in the lungs did not differ between P4- and placebo-treated females (Table 1). These data indicate that P4 did not affect the cell-mediated antiviral immune response during acute IAV infection.

**Table 1:** Total numbers of CD4+ and CD8+ T cells in lung single cell suspensions from IAV-infected ovariectomized female mice treated with placebo (-P4) or progesterone (+P4) at 7dpi.

Total numbers of cells	-P4	+P4
CD8+ T cells (x10^5^)	2.43±0.52	1.91±0.29
NP_366-374_ CD8+ T cells (x10^4^)	5.7±0.49	3.28±0.68
IFN-γ+ CD8+ T cells (x10^4^)	1.37±0.6	1.84±0.65
TNF-α CD8+ T cells (x10^4^)	7.44±2.2	5.42±1.76
Total CD4+ T cells (x10^5^)	4.35±0.73	3.46±0.52
IFN-γ+ CD4+ T cells (x10^4^)	2.96±0.91	3.45±1.22
IL4+ CD4+ T cells (x10^4^)	2.62±0.75	5.14±1.76
Foxp3+ CD4+ T cells (x10^4^)	5.58±2.28	3.47±1.66

Data are presented as the mean ± SEM from four independent experiments (CD8+ T cells: n = 6-8/treatment; CD4+ T cells: n = 10-12/treatment) and were analyzed by T-tests.

### Progesterone promotes a repair environment in the lungs during lethal IAV infection

**Fig 2:**
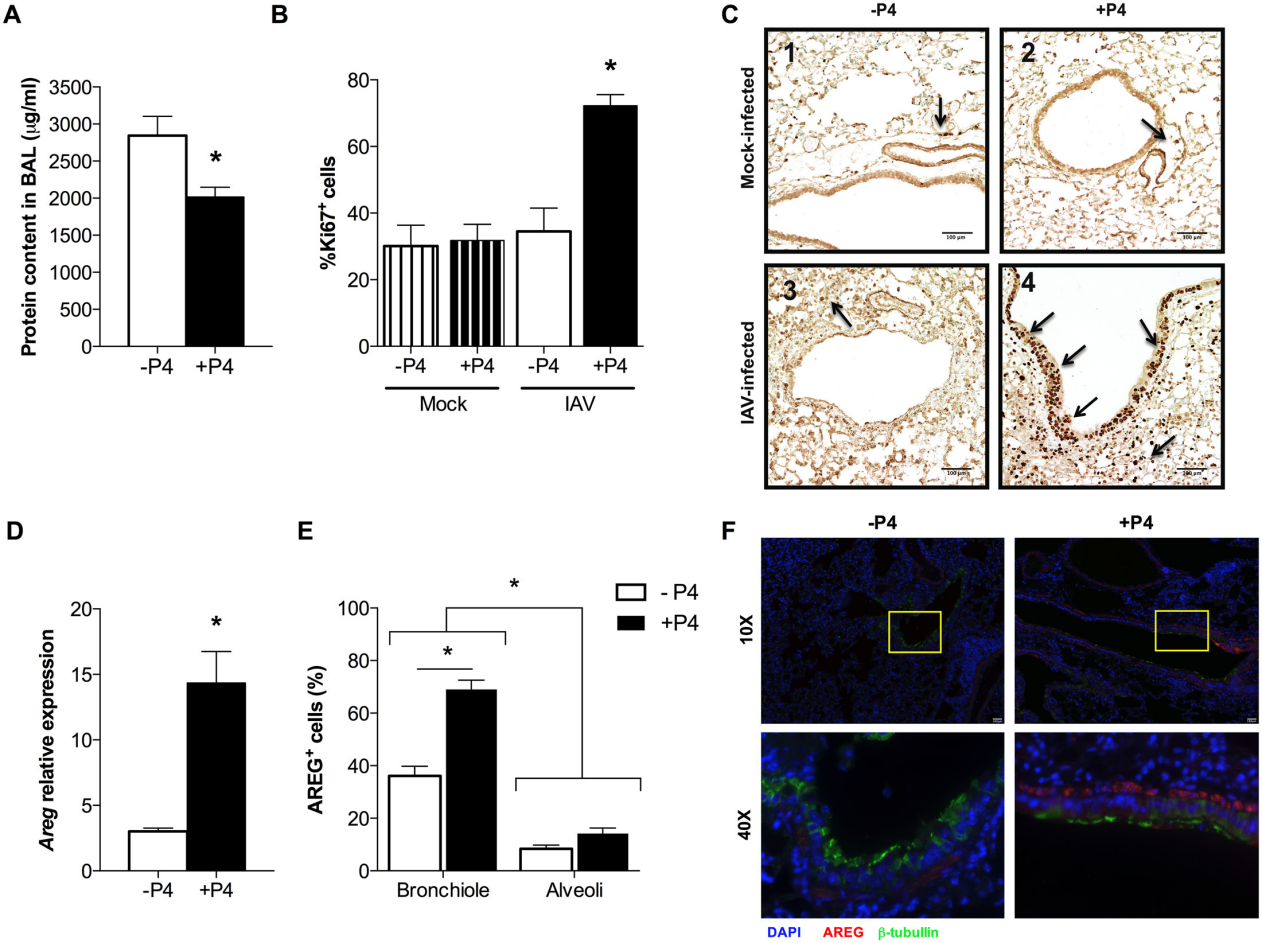
Progesterone (P4) treatment promotes barrier integrity, cellular proliferation, and induction of amphiregulin (AREG) in the lungs of IAV-infected female mice. Adult female mice were ovariectomized, treated with placebo (-P4) or exogenous P4 (+P4), and inoculated with lethal IAV or mock-infected. At peak disease (7dpi), bronchoalveolar lavage (BAL) fluid or whole lungs were harvested or fixed for histology. Total protein content in the BAL was measured by BCA assay (A). Cellular proliferation was assessed at 7 dpi using the marker Ki67 in paraffin-embedded lung tissue sections; sections were counterstained with hematoxylin; and arrows indicate examples of Ki67+ cells (C). The percentages of Ki67+ cells were analyzed and quantified using ImageJ (B and C; 10X magnification). *Areg* mRNA expression was quantified and normalized to *Gapdh* and to mock-infected controls (D). The percentages of AREG+ cells (red) in bronchioles and alveolar airspace were analyzed using immunofluorescence and quantified using ImageJ (E; n = 20 fields/treatment). Representative images of bronchioles (10X magnification) and focused areas (40X magnification) with epithelial cells (β-tubulin^+^ cells, in green) are shown (F). Bars represent means ±SEM from two or three independent experiments. Significant differences are represented by an asterisk (*) (mock: n = 6; IAV: n = 10–12).

IAV infection is characterized by the induction of a cytokine storm and excessive immunopathology, which leads to tissue damage [37]. Damage to the lung endothelium and/or epithelium results in vascular leakage into the air spaces, and can be quantified by measuring protein concentration in bronchoalveolar lavage (BAL) fluid. Consistent with the histopathological findings of increased vasculitis and edema (Fig 1H) following lethal IAV infection, treatment of females with P4 decreased the total amount of protein contained in the BAL as compared to placebo-treated mice (Fig 2A; *P*<0.05). Among infected females, treatment with P4 also increased cellular proliferation (as measured by Ki67 expression) in the lungs as compared to placebo treatment during peak disease (7dpi) (Fig 2B and 2C; *P*<0.05). Analysis of the expression of Ki67 in the different areas of the lungs revealed greater proliferation in several regions of the lungs, but was most pronounced in the epithelial cells lining the airways during IAV infection in P4-treated mice (Fig 2C). The epidermal growth factor, AREG, promotes proliferation of epithelial cells and protects mice from excessive pathology during IAV infection [18, 19]. Analysis of AREG expression during peak disease (7 dpi) revealed increased mRNA expression, as well as AREG protein in the bronchioles, but not the alveoli, in the lungs of P4-treated mice as compared to placebo-treated mice infected with IAV (Fig 2D–2F, *P*<0.05).

### Progesterone induces Th17 cells in the lungs of IAV-infected female mice

**Fig 3:**
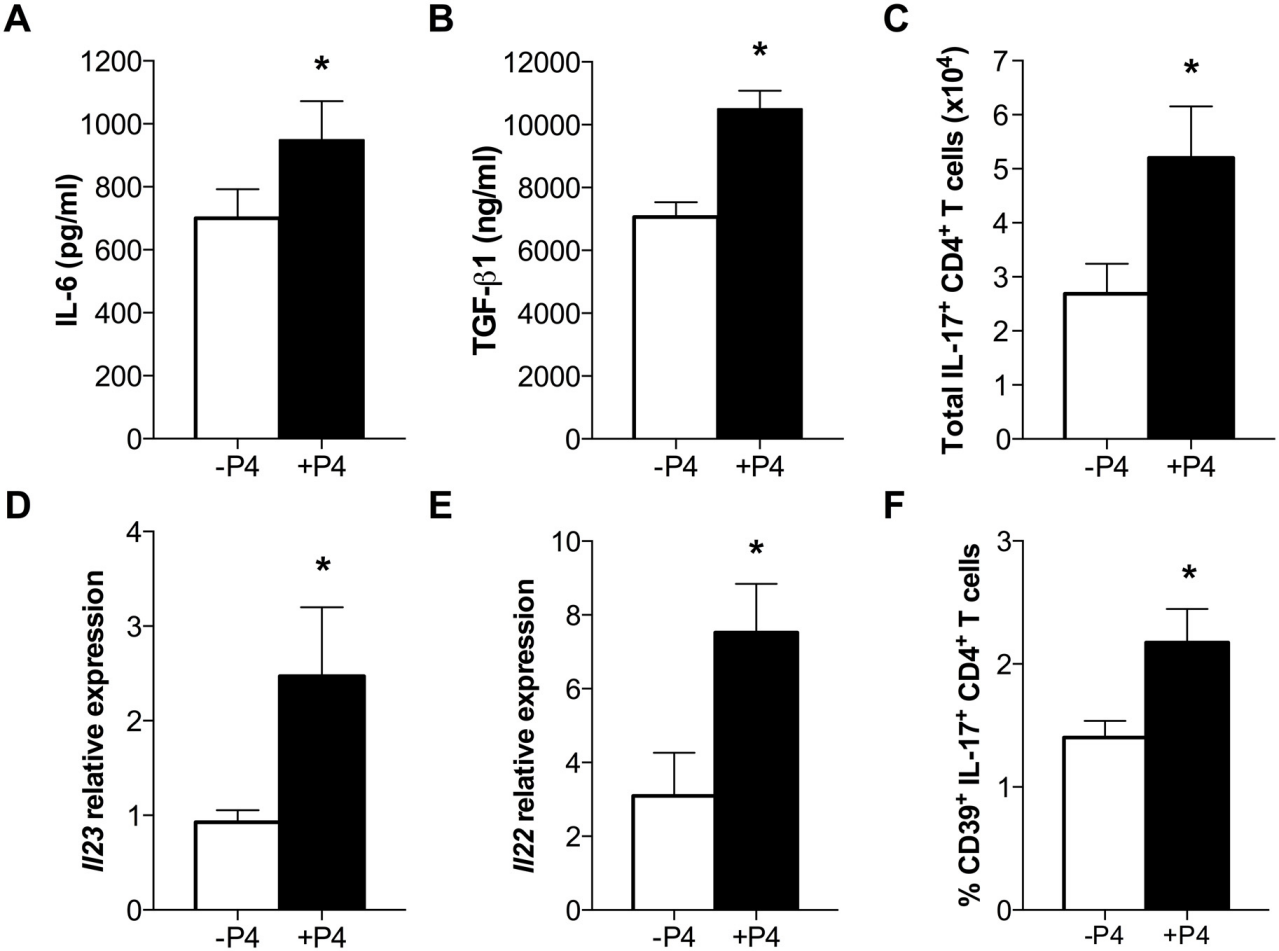
Progesterone (P4) treatment induces regulatory Th17 cells in the lungs of IAV-infected female mice. Adult female mice were ovariectomized, treated with placebo (-P4) or exogenous P4 (+P4), and inoculated with lethal IAV or mock-infected. At peak disease (7 dpi), supernatant from whole lung homogenates was used to quantify IL-6 (A) and TGF-β (B). The total numbers of Th17 (C) cells were measured by flow cytometry in lung single-cell suspensions stimulated *ex vivo* with IAV-specific antigen (D^b^HA_211-255_ and D^b^NP_311-325_) in presence of BFA. The expression of *Il23* and *Il22* was analyzed in lung tissue and normalized to GAPDH and to mock-infected controls using the ΔΔCt method (D and E). Expression of CD39 was evaluated by flow cytometry on Th17 cells (F) from lung single-cell suspensions. Bars represent means ±SEM from two or three independent experiments. Significant differences are represented by an asterisk (*) (mock: n = 6; IAV: n = 10–12).

Progesterone treatment altered inflammation during IAV infection (Fig 1G and 1H) and induced a repair environment through cellular proliferation and restoration of barrier integrity (Fig 2A–2C). To further characterize the effect of P4 on inflammatory responses to IAV, a panel of 13 cytokines and chemokines was analyzed in the supernatant of whole lung homogenates. As expected, following infection with IAV, pulmonary concentrations of IL-1β, TNF-α, IFN-γ, and IL-12p70 were significantly increased during the first week of infection in all females, regardless of P4 treatment (S1 Table; *P*<0.05). P4 treatment decreased pulmonary production of the alarmins IL-13 and IL-33 as compared with placebo treatment during IAV infection (S1 Table; *P*<0.05). The only two cytokines that were significantly increased in P4-treated females compared with placebo-treated females during IAV infection were IL-6 and TGF-β (Fig 3A and 3B; *P*<0.05). P4 treatment of IAV-infected mice had no effect on the other canonical regulatory protein, IL-10, as compared to placebo treatment (S1 Table).

Production of TGF-β and IL-6 increases differentiation of Th17 cells. Th17 cells promote repair of the gut epithelium [38] and may be similarly involved in orchestrating repair of the pulmonary epithelium. To test this hypothesis, populations of CD4+ T cells from mock- and IAV-infected mice were enumerated during peak disease (7 dpi). There was no effect of P4 treatment on total numbers of CD4+ T cells, Th1, Th2, or Treg cells in the lungs at 7 dpi (Table 1). In contrast, P4 treatment increased the total number of Th17 cells in the lungs during IAV infection as compared with placebo treatment (Fig 3C; *P*<0.05). The cytokine IL-23 is necessary for maintenance of Th17 cells and the expression of *Il23* mRNA in the lungs was increased in P4- compared with placebo-treated females (Fig 3D; *P*<0.05). Th17 cells exert their tissue reparative effects by increasing the production of IL-22 [39]. The expression of *Il22* mRNA in the lungs was greater in P4- than placebo-treated females during IAV infection (Fig 3E; *P*<0.05). Finally, one surface marker on Th17 cells that is associated with reducing inflammation (i.e., regulatory or suppressive Th17 cells) is the ectonucleotidase CD39 (ref. [40, 41]). The percentage of Th17 cells that expressed CD39 was significantly increased in P4-treated as compared to placebo-treated females during IAV infection (Fig 3F; *P*<0.05). These data indicate that P4 alters the inflammatory milieu of the lungs by promoting a repair environment in IAV-infected female mice, with increased numbers of regulatory Th17 cells, elevated expression of *Il22*, and upregulated expression of *Areg* during lethal IAV infection.

### Progesterone accelerates long-term pulmonary recovery during sublethal IAV infection

**Fig 4:**
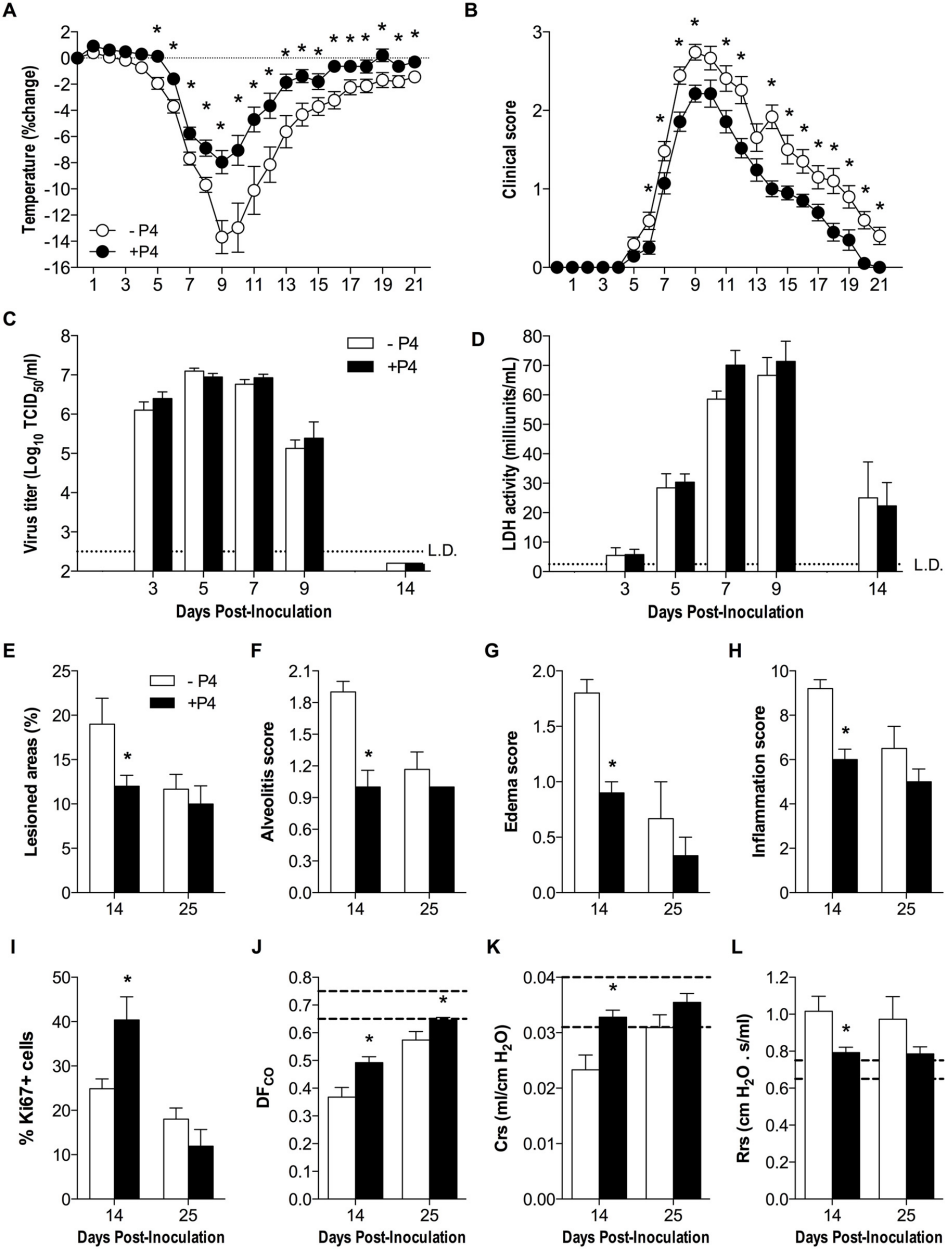
Progesterone (P4) reduces inflammation and improves pulmonary function during sublethal IAV infection. Adult female mice were ovariectomized, treated with placebo (-P4) or exogenous P4 (+P4), and inoculated with a sublethal dose of IAV or mock-infected. Females (n = 23-25/treatment) were monitored daily for changes in rectal body temperature (A) and clinical disease (B) for 21 dpi. Infectious virus titers (C) and cell necrosis (D) were measured 3, 5, 7, 9 and 14 dpi (n = 5–10 per dpi). Percentage of lesioned areas (E), alveolitis scores (F), edema scores (G), and cumulative inflammation scores (H) were quantified in H&E stained lung sections at 14 and 25 dpi. The numbers of proliferating of Ki67+ cells were analyzed at 14 and 25 dpi and quantified using ImageJ (I) (n = 3-5/treatment/dpi with 10 fields per animal). Pulmonary function tests, measuring lung diffusing capacity (DF_CO_; J), lung tissue compliance (Crs; K), and resistance (Rrs; L), were performed at 14 and 25 dpi with the dotted line representing the average value (mean ±SEM) for mock-infected mice (n = 7-10/treatment/dpi). Data represent means ±SEM from 2–3 independent experiments and significant differences are represented by asterisks (*).

To further evaluate the role of P4 in lung repair and recovery from IAV infection, P4- and placebo-treated female mice were infected with a less pathogenic IAV strain, ma2009, at a dose (0.4mLD_50_) that allowed for monitoring of the mice over a longer duration of time. Similar to lethal IAV infection, P4-treated females infected with sublethal IAV showed less hypothermia (Fig 4A; *P*<0.05) and reduced clinical disease (Fig 4B; *P*<0.05) as compared to placebo-treated females. Analysis of pulmonary virus titers confirmed that P4 did not alter virus titers or clearance of infectious virus over the course of IAV infection (Fig 4C). To determine if P4 reduced cell death due to IAV infection, LDH levels in the BAL fluid were quantified. Cellular damage during IAV infection was not altered by treatment with P4 as compared with placebo (Fig 4D). Lung sections were evaluated for markers of inflammation and damage during the recovery (14 dpi) and post-recovery (25 dpi) phases of IAV infection. At 14 dpi, but not at 25 dpi, treatment of IAV-infected female mice with P4 decreased the percentage of lesioned areas, alveolitis, edema, and cumulative inflammation as compared to placebo-treated mice (Fig 4E–4H, *P*<0.05). Treatment with P4 significantly increased Ki67 expression in pulmonary cells during the recovery phase (14 dpi) of IAV infection as compared with placebo treatment (Fig 4I; *P*<0.05). Based on the observation that P4 treatment promoted lung repair in IAV-infected female mice, we evaluated the impact of P4 on overall lung physiology during (14 dpi) and after (25 dpi) recovery from sublethal IAV infection by assessing markers of pulmonary function. Lung diffusing capacity (DF_CO_), lung tissue compliance (Crs), and resistance (Rrs) returned to baseline faster in P4- than placebo-treated mice infected with IAV (Fig 4J–4L, *P*<0.05). Treatment of female mice with P4 reduces inflammation and promotes faster recovery from sublethal IAV infection.

### The protective effects of P4 against influenza are mediated by AREG

**Fig 5:**
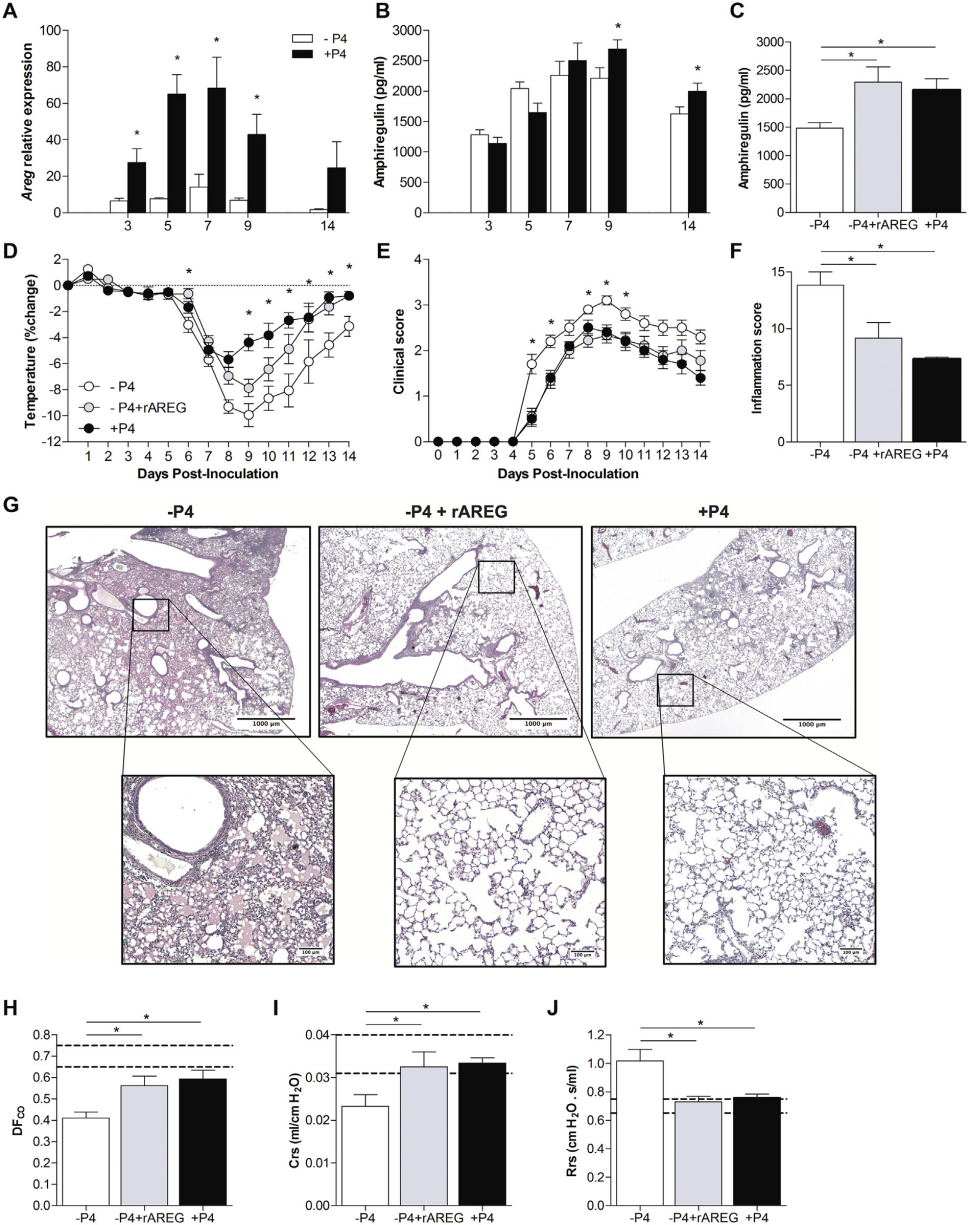
Progesterone (P4) increases amphiregulin (AREG) expression and administration of recombinant AREG protects P4-depleted female mice against IAV infection. Adult female mice were ovariectomized, treated with placebo (-P4) or exogenous P4 (+P4), and inoculated with a sublethal dose of IAV or mock-infected. The expression of amphiregulin (*Areg*) mRNA (A) and protein concentrations (B) in the lungs were quantified at 3, 5, 7, 9 and 14 dpi (n = 8-10/treatment/dpi). Gene expression was normalized to *Gapdh* and mock-infected controls using the ΔΔCt method. Ovariectomized mice were treated with placebo (-P4), placebo and recombinant amphiregulin (-P4 +rAREG), or P4 (+P4) and inoculated with a sublethal dose of IAV. To confirm AREG replacement, pulmonary concentrations of AREG were measured at 14 dpi (C). Mice were monitored daily for changes in body temperature (D) and clinical disease (E) (n = 9-10/treatment). H&E stained lung sections collected at 14 dpi were scored for inflammation as a cumulative score of perivasculitis, vasculitis, bronchiolitis, alveolitis, edema, consolidation, and necrosis (F). Representative images of overall inflammation (2X magnification) and focused areas (10X magnification) with cellular infiltration and edema are shown (G) (n = 3-5/treatment, with 10 fields per animal). Pulmonary function tests were performed at 14 dpi and lung diffusing capacity (DF_CO_; H), lung compliance (Crs; I), and resistance (Rrs; J) were measured (n = 8-10/treatment). The dotted lines represent the value (means ±SEM) for mock-infected mice and bars and circles represent means ±SEM for IAV-infected mice from 2 independent experiments, with significant differences represented by asterisks (*).

Progesterone increased pulmonary AREG expression during lethal IAV infection (Fig 2D–2F) and increased AREG expression is associated with an improved outcome from lethal IAV infection [18, 19]. In our sublethal IAV model, we were able to measure pulmonary expression and production of AREG over a longer duration of time to establish the effects of P4 on the kinetics of AREG production in females. P4-treatment induced a 30–70 fold greater induction of *Areg* mRNA and higher concentrations of AREG protein in the lungs as compared with placebo treatment over the course of IAV infection (Fig 5A and 5B; *P*<0.05). Peak production of AREG occurred at 9 dpi (Fig 5B), which corresponded with peak disease (Fig 4A and 4B) during sublethal IAV infection. To test the hypothesis that reduced AREG production in P4-depleted females caused a more severe outcome from IAV, we treated P4-depleted female mice with recombinant AREG (rAREG) during the course of IAV infection. Treatment of P4-depleted mice with rAREG resulted in AREG levels that were comparable to those of P4-treated mice at 14 dpi (Fig 5C; *P*<0.05). Treatment of P4-depleted females with rAREG significantly improved the recovery from IAV infection (Fig 5D and 5E; *P*<0.05), with reduced inflammation (Fig 5F and 5G; *P*<0.05) and improved pulmonary function, including lung diffusing capacity (DF_CO_), lung compliance (Crs), and resistance (Rrs), to levels similar to that of P4-treated females (Fig 5H–5J; *P*<0.05). These data suggest that the protective effects of P4 on IAV disease may be mediated by an upregulation of AREG.

**Fig 6:**
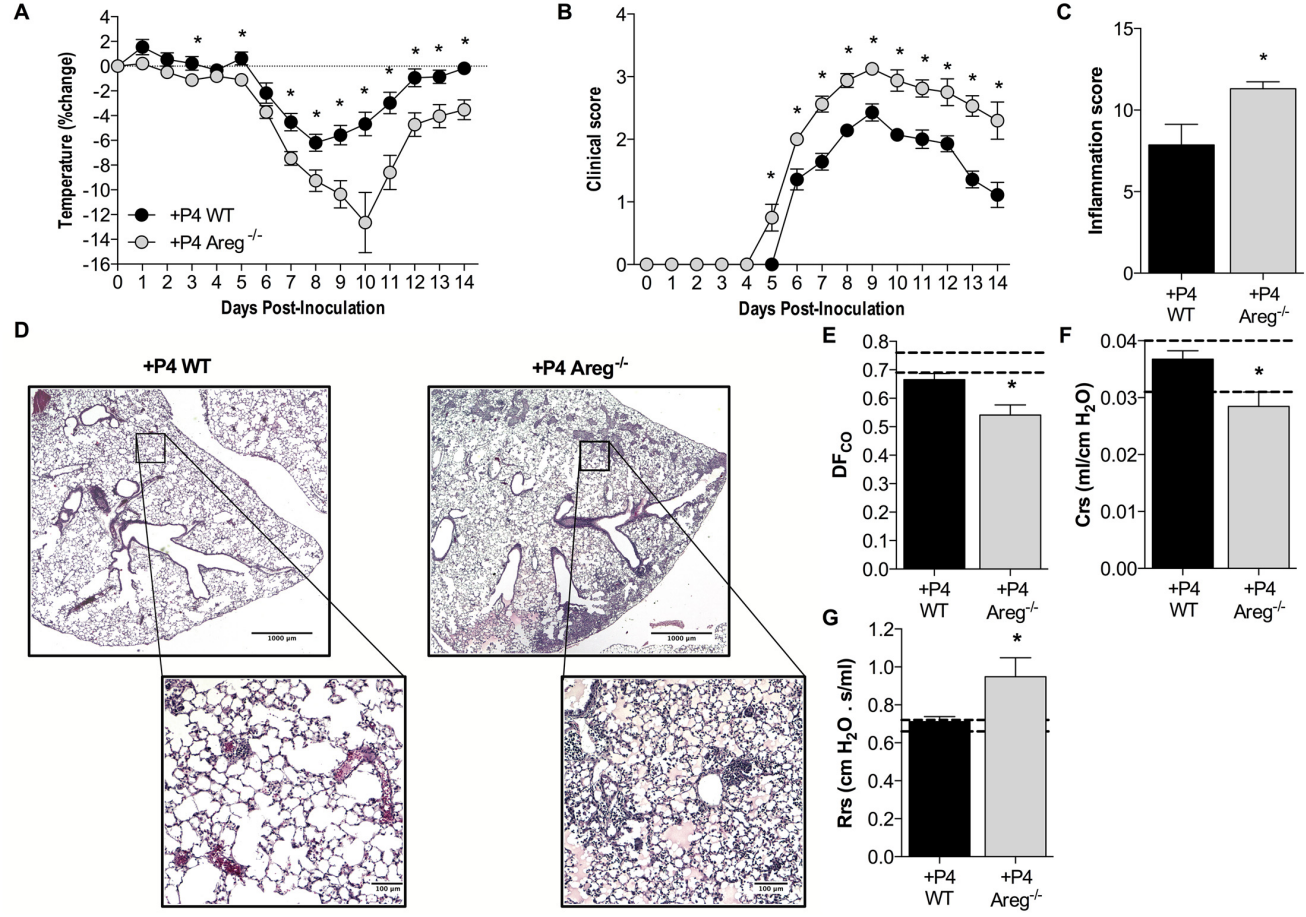
Deletion of amphiregulin (*Areg*) reverses the protective effects of progesterone (P4) during IAV infection. Female *Areg* ^-/-^ or WT littermates were ovariectomized, treated with P4, inoculated with a sublethal dose of IAV or mock-infected, and monitored daily for changes in body temperature (A) and clinical disease (B) (n = 15/treatment). At 14 dpi, inflammation was scored from H&E stained lung sections for inflammation as a cumulative score of perivasculitis, vasculitis, bronchiolitis, alveolitis, edema, consolidation and necrosis (C). Representative images of overall inflammation (2X magnification) and focused areas (10X magnification) with cellular infiltration and edema are shown (D) (n = 5/treatment, with 10 fields per animal). Pulmonary function tests were performed at 14 dpi and lung diffusing capacity (DF_CO_; E), lung compliance (Crs; F), and resistance (Rrs; G) were measured (n = 10-12/treatment). The dotted lines represent the value (means ±SEM) for mock-infected Areg-/- mice and bars and circles represent means ±SEM for IAV-infected mice from 2 independent experiments, with significant differences represented by asterisks (*).

The contribution of AREG to P4-mediated protection from IAV infection was further determined by using mice that lacked the expression of a functional *Areg* gene [42]. Deletion of the *Areg* gene in female mice (*Areg* ^*-/-*^) reversed the protective effects of P4 on the outcome of IAV infection (Fig 6A and 6B; *P*<0.05). This was accompanied by increased inflammation in P4-treated *Areg-/-* as compared with WT female mice (Fig 6C and 6D; *P*<0.05). Improvement of pulmonary function in the presence of P4, as measured by lung diffusing capacity (DF_CO_), compliance (Crs), and resistance (Rrs), was also reversed in IAV-infected *Areg* ^*-/-*^ mice as compared with WT mice treated with P4 (Fig 6E–6G; *P*<0.05). Taken together, these data indicate that P4 treatment of IAV-infected female mice promotes a pulmonary repair environment and restoration of lung function through the induction of AREG.

### Progesterone accelerates wound healing and increases production of AREG by respiratory epithelial cells

**Fig 7:**
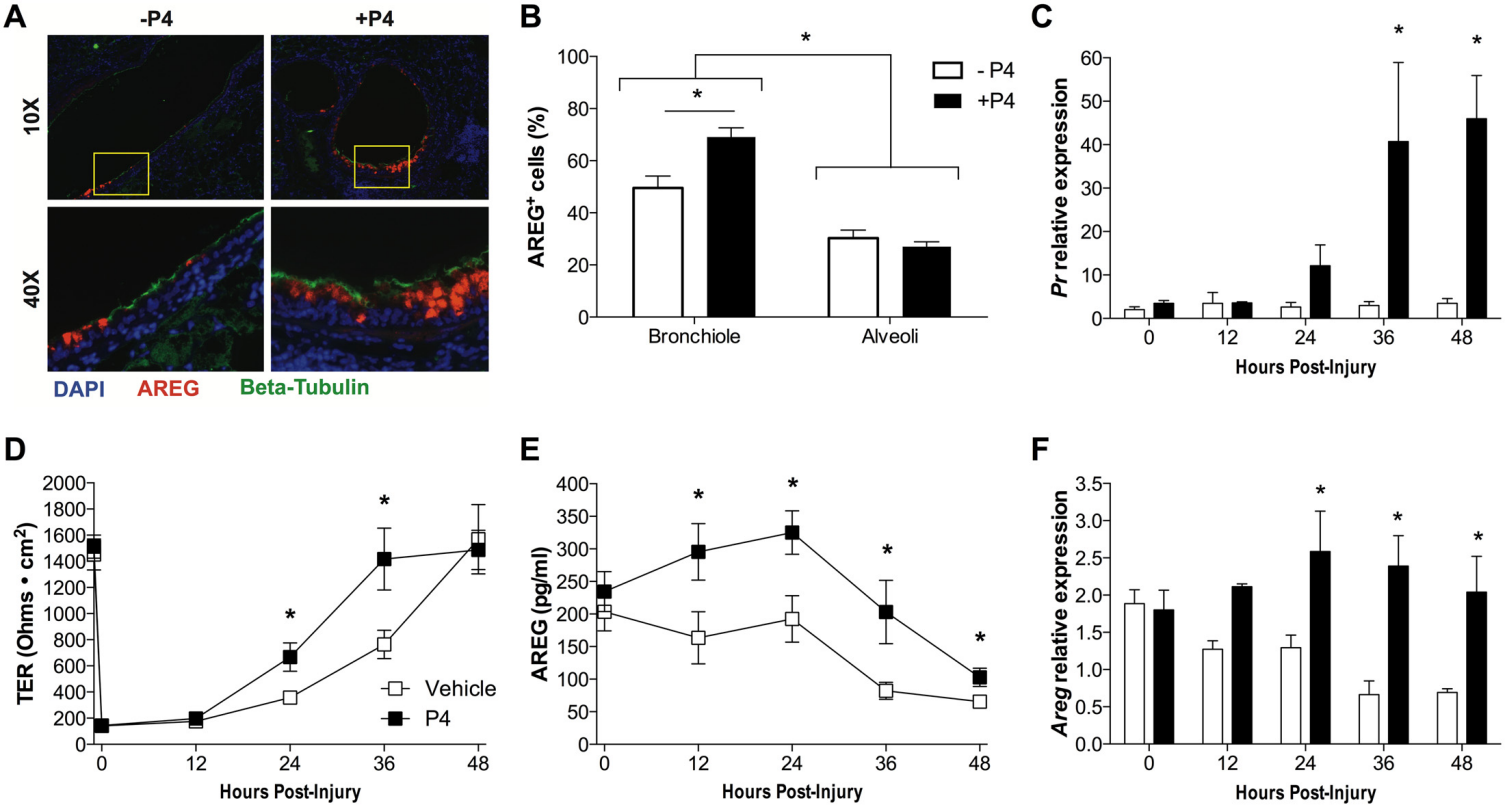
Progesterone induces amphiregulin (AREG) and accelerates wound healing in respiratory epithelial cells. Adult female mice were ovariectomized, treated with placebo (-P4) or exogenous P4 (+P4), and inoculated with a sublethal dose of IAV. Animals were euthanized at 14 dpi and lungs were fixed for histology (n = 5/treatment). AREG expression (in red) was assessed in epithelial cells (β-tubulin^+^ cells, in green) using immunofluorescence in different areas of the lung tissue (A). Representative images of bronchioles (10X magnification) and focused areas (40X magnification) are shown. The percentages of AREG+ cells in bronchioles and alveolar airspace were analyzed and quantified using ImageJ (B; n = 20 fields/treatment). Mouse tracheal epithelial cell (mTEC) cultures were treated with vehicle (EtOH) or P4 and injured or left intact. Relative expression of progesterone receptor (*Pr*) mRNA was measured every 12 h for 48h (C). Transepithelial resistance (TER) (D), AREG production (E), and *Areg* mRNA relative expression (F) were measured every 12h for 48h (n = 10/treatment/time-point). *Areg* and *Pr* mRNA expression was normalized to *Gapdh* and to uninjured controls using the ΔΔCt method (n = 5/treatment/time-point). Bars or squares represent means ±SEM from 2–3 independent experiments, with significant differences represented by asterisks (*).

Treatment with P4 induces higher expression of AREG in the lungs of sublethal IAV-infected females, particularly in the epithelial cells lining the larger airways, as compared with placebo-treatment (Fig 7A and 7B; *P*<0.05). To assess the contribution of P4 treatment to the repair of damaged respiratory epithelia, we used an *in vitro* model system in which primary, differentiated mouse tracheal epithelial cell (mTECs) cultures were mechanically injured. The mTECs express the progesterone receptor (*Pr*), which was upregulated in the presence of P4 (Fig 7C; *P*<0.05). Repair of the epithelial cell layer was measured over time to identify the return of the transepithelial resistance (TER) to baseline. Following injury, cultures of mTECs treated with P4 returned to baseline TER faster than vehicle-treated cultures (Fig 7D; *P*<0.05). During injury, mTEC cultures treated with P4 produced more AREG mRNA and protein than vehicle-treated mTECs cultures (Fig 7E and 7F; *P*<0.05). These data illustrate that P4 improves pulmonary repair and function by increasing AREG production and wound repair in epithelial cells.

## Discussion

Hosts have evolved several mechanisms for overcoming viral infections, such as the induction of antiviral defenses that increase resistance to infection, or the activation of regulatory and repair responses that increase tolerance to the negative consequences of infection. In the present study, P4 significantly protected females during IAV infection by altering inflammation, improving pulmonary function, and promoting a pulmonary repair environment, which resulted in an earlier recovery. The protective effects of P4 were primarily mediated by the induction of AREG during both lethal and sublethal infections. Progesterone did not increase resistance to infection in females as demonstrated by the lack of an effect of P4 treatment on virus titers, clearance of infectious virus, numbers of Th1 cells, and CD8+ T cell activity in lungs. Instead, P4 reduced the detrimental consequences of IAV infection in females by increasing their tolerance to infection. Several host immunological factors, including TGF-β, Tregs, and regulatory populations of CD39+ Th17 cells, are associated with maintaining the balance between protective and pathological immune responses during IAV infection. Although P4 treatment had no effect on the numbers of Tregs in the lungs during IAV infection, concentrations of TGF-β and IL-6, the expression of *Il23* and *Il22*, the number of Th17 cells, as well as the proportion of Th17 cells expressing CD39, were increased. Regulatory Th17 cells express the ectonucleotidase CD39 and are associated with repair following inflammation and infection [40, 41]. Th17 cells also promote epithelial cell proliferation and repair in the gut, primarily through the induction of IL-22 [38]. Consequently, treatment of females with P4 increased IL-22, a cytokine that has been shown to mediate regeneration of lung epithelial cells following IAV infection [43]. Whether the P4-induced increase in regulatory Th17 cells and IL-22 promotes cellular proliferation and repair of the lung epithelium during IAV infection by increasing AREG production requires consideration. Because P4 directly induced AREG production in respiratory epithelial cells *in vitro*, P4-induced AREG production may occur independent of the reparative effects of regulatory Th17 cells in the lungs during IAV infection.

Progesterone induces repair of epithelial cells in the endometrium and myelin fibers in the central nervous system [44, 45]. This repair of myelin fibers by P4 [46] is one factor mediating how this reproductive hormone mitigates the progression of multiple sclerosis [29]. Our data show that P4 promotes proliferation of pulmonary cells, including epithelial cells, and pulmonary tissue repair. The reparative effects of P4 in the reproductive tract are caused by the induction of AREG, which promotes epithelial remodeling in mammary and uterine tissues [22, 23]. In the respiratory tract, AREG is involved in pulmonary tissue remodeling and repair during lung injury, asthma, and infection [18, 19, 21, 47, 48]. Although *Areg*-gene deficient mice show few abnormalities under homeostatic conditions [42], their ability to resolve inflammation or infection is severely impaired [20, 21]. During IAV infection, administration of rAREG protects mice from severe IAV-mediated morbidity by decreasing hypothermia, improving pulmonary function, and decreasing protein leakage into the airways [18, 19]. The data presented are the first report of P4 induction of AREG outside of the reproductive tract and in the context of infection. The effect of other reproductive hormones on AREG expression, including differential expression between males and females, warrants further study.

AREG is produced primarily by epithelial cells [49], but type 2 innate lymphoid cells (ILC2) and Tregs have also been shown to produce AREG during IAV infection and contribute to the repair during resolution of infection [18, 19, 49, 50]. Because each of these cell type express progesterone receptors [5, 51], each is a potential producer of AREG in response to P4 treatment. Our *in vivo* and *in vitro* data suggest that respiratory epithelial cells are a predominant source of P4-induced AREG. Following IAV infection, AREG expression was predominantly localized to the bronchiolar epithelial cells, and P4 treatment of isolated mTECs increased AREG production following mechanical damage. Furthermore, P4-treatment did not activate markers of ILC2s, including IL-13 and IL-33 production, or increase numbers of Tregs in the lungs during infection, suggesting that the induction of AREG in response to P4 may not be occurring in these immune cell populations.

Recovery following IAV infection is generally defined as a return of body temperature or body mass back to homeostatic levels [52]. In this study, however, we showed that pulmonary pathology and impaired pulmonary function persisted after measures of overall health, including hypothermia and clinical disease, returned to baseline. Furthermore, the impact of IAV infection was observed long after infectious virus had been cleared from the lungs. Recovery following IAV infection extended beyond 21 dpi and should be defined not only by reduced morbidity, but also by restored pulmonary function, both of which were expedited by P4 treatment in females.

Progesterone concentrations fluctuate naturally during the female life span, with moderate concentrations during the menstrual cycle, high concentrations during pregnancy, and low concentrations following menopause. Progesterone is also used exogenously by over 100 million women worldwide in P4-based hormonal contraceptives, by post-menopausal women taking hormonal replacement therapy, and by both men and women in the treatment of cancer, osteoporosis, and brain injury [3, 53]. Prior to this study, the health consequences of P4-based therapies in acute respiratory infection had not been characterized. We have demonstrated that AREG, which is a significant factor that induces tissue repair and recovery from infectious diseases, is regulated by P4 during both lethal and sublethal IAV infection. The data presented provide critical mechanistic information about how P4 and possibly synthetic P4 analogues affect women’s health outside of the reproductive tract. Contraceptives that contain P4 are listed as an essential medication by the WHO, being a safe and effective method for improving health outcomes in women, including those living with HIV [1]. During outbreaks of infectious diseases that harm pregnant women and their fetuses (e.g., the current Zika outbreak), the WHO recommends increased use of hormonal contraceptives, which according to our data could have additional beneficial consequences on the outcome of other infectious diseases.

## Materials and Methods

### Ethics statement

All experiments were performed in compliance with the standards outlined in the National Research Council’s Guide to the Care and Use of Laboratory Animals. The animal protocol (M015H236) was reviewed and approved by the Johns Hopkins University Animal Care and Use Committee. All efforts were made to minimize animal suffering.

### Animals

Adult (7–8 weeks old) female C57BL/6 mice were purchased from NCI Frederick. *Areg* ^+/-^ (C57BL/6 129 Sv) mice were kindly provided by Dr. Marco Conti (University of California San Francisco) and bred to obtain *Areg* ^*-/-*^ and *Areg* ^*+/+*^ female littermates. Mice were housed 5 per microisolator cages under standard BSL-2 housing condition with food and water ad libitum.

### Surgical procedures

At 8–12 weeks of age, mice were anesthetized with an intramuscular injection of ketamine (80 mg/kg) and xylazine (8 mg/kg) cocktail and ovaries were removed bilaterally as previously described [25]. All animals were given two weeks to recover prior to infection. Recombinant amphiregulin (10μg; R&D) was administered intraperitoneally every other day using saline as the vehicle.

### Hormone replacement and quantification

Ovariectomized (ovx) mice were assigned to receive subcutaneous implants of placebo (-P4) or 15 mg progesterone (+P4) 21-day release pellets (Innovative Research of America) prior to IAV inoculation. Circulating concentrations of P4 were assessed from plasma using ether extraction and radiolabelled immunoassay, with P4 antibody (MP Biomedicals) and tracer 3H-P4 (American Radiolabeled). Uterine horns were removed at several time-points upon euthanasia of mice and wet weight was quantified as a bioassay for P4.

### Virus infection and quantification

Mouse-adapted influenza A viruses, A/Puerto Rico/8/34 (PR8; H1N1) provided by Dr. Maryna Eichelberger at the Food and Drug Administration (FDA) and A/California/04/09 (ma2009; H1N1) generated by Dr. Andrew Pekosz from a published sequence [54], were used in these studies. Mice were anesthetized and inoculated intranasally with 30 μl of DMEM (mock) or H1N1 virus (1.78 50% mouse lethal dose (MLD_50_) for PR8 and 0.4 MLD_50_ for ma2009). Clinical disease scores for IAV-infected mice were based on four parameters, with one point given for each of the following: dyspnea, piloerection, hunched posture and absence of an escape response. For virus quantification, log_10_ dilutions of lung homogenates (starting at 10^−1^) were plated onto a monolayer of MDCK cells in replicates of 6 for 4–6 days. Cells were stained with naphthol blue black (Sigma Aldrich) and scored for cytopathic effects. The 50% tissue culture infectious dose (TCID_50_) was calculated according to the Reed-Muench method.

### Cytokine and chemokine quantification

Snap-frozen lung tissue was homogenized in DMEM supplemented with 1% penicillin/streptomycin and 1% L-glutamine (Invitrogen) and centrifuged to remove cellular debris. Supernatants were harvested to measure IL-1β, TGF-β, IL-4, IL-5, IL-13, IL-17, IL-33, and AREG by ELISA (R&D Systems and BD Biosciences) and CCL-2, IL-12(p70), TNF-α, IFN-γ, IL-6 and IL-10 with the mouse inflammation cytometric bead array (BD Biosciences) according to the manufacturer’s protocols.

### Real time reverse transcription PCR

Snap-frozen lung tissue or mTECs were homogenized in TRIzol and RNA was purified by chloroform extraction. RNA concentration and purity was measured using a NanoDrop (ThermoFisher Scientific). The RNA concentration in each sample was standardized to 1 μg using RNAse-free water. Reverse transcription was carried out using the iScript cDNA synthesis kit (Biorad) according to the manufacturer’s protocol. Pre-designed *Il23* (Mm.PT.58.10594618.g), *Il22 (NM_016971*.*2)*, *Areg* (Mm.PT58.31037760), *Gapdh* (Mm.PT.39a.1) and *Pr (*Mm.PT.58.10254276*)* PrimeTime Primers were purchased from Integrated DNA Technologies. Semi-quantitative RT-PCR was performed in 96-well optical reaction plates using the SsoFast EvaGreen Supermix (Biorad) on the StepOnePlus RT-PCR system (Applied Biosystems). Gene expression was normalized to *Gapdh* and mock-infected samples or wells with no injury using the ΔΔCt method.

### Flow cytometry analyses of T cells

Lungs were excised and single-cell suspensions were generated following red blood cell lysis. Total viable cells were determined using a hemocytometer and trypan blue (Invitrogen) exclusion and resuspended at 1x10^6^ cells/ml in RPMI 1640 (Cellgro) supplemented with 10% FBS (Fisher Scientific) and 1% penicillin/streptomycin. For IAV-specific T cells enumeration, cells were cultured for 5h with IAV peptide antigen (CD8:NP_366-374_, or CD4: HA_211-255_, NP_311-325,_ respectively) (ProImmune) in media containing Brefeldin A (GolgiPlug, BD) The viability of cells was determined by fixable Live/Dead violet viability dye (Invitrogen) and Fc receptors were blocked using anti-CD16/32^A^. The T cell populations were stained with the following antibodies: PerCP-Cy5.5 conjugated anti-CD4 (RM4-5)^A^, PerCP-Cy5.5 conjugated anti-CD8 (53–6.7)^A^, FITC conjugated anti-CD25 (7D4)^A^, PE conjugated D^b^NP_366-374_ tetramer (NIH Tetramer Core Facility), FITC conjugated anti-CD4 (RM4-5)^B^, APC conjugated anti-CD3 (17A2^B^, and PerCP-eFluor 710 conjugated anti-CD39 (24DMS1)^B^. Intracellular staining with PE conjugated anti-TNF-α(MP6-XT22)^A^, FITC conjugated anti-IFN-γ (XMG1.2)^A^, PE conjugated anti-IL-4 (11B11)^A^, and PE conjugated anti-IL-17 (TC11-1810)^A^, was performed following permeabilization and fixation with Cytofix/Cytoperm and Perm/Wash buffer^A^. Intracellular staining with PE-conjugated Foxp3 (MF23)^A^ was performed following fixation and permeabilization with a Foxp3 staining buffer set^A^. Data were acquired using a FACS Calibur (Cellquest Software) and analyzed using FlowJo (Tree Star, Inc.). Total cell counts were determined by multiplying each live cell population percentage by the total live cell counts acquired prior to staining by trypan blue exclusion counts on a hemocytometer. All reagents were purchased from BD Biosciences^A^ or eBioscience^B^ unless stated otherwise.

### Histopathology and immunohistochemistry

Lungs were inflated, fixed in Z-fix (Anatech), embedded in paraffin, cut into 5μm sections, and mounted on glass slides. Slides were stained with hematoxylin and eosin (H&E) and used to evaluate lung inflammation. Histopathological scoring was performed by a single blinded veterinary pathologist on a scale from 0–3 (0, no inflammation; 1, mild inflammation; 2, moderate inflammation; and 3, severe inflammation) for the following parameters: bronchiolitis, alveolitis, vasculitis, perivasculitis, necrosis, consolidation, and edema [55, 56]. The sum of these parameters represents the cumulative inflammation score. The percentage of lesioned areas within each tissue section was also evaluated. Histopathological slides were deparafinized with xylene and rehydrated in graded ethanol. Heat-induced antigen retrieval with citrate buffer was performed and slides were blocked with 10% normal serum prior to overnight primary antibody incubation. For Ki67, rabbit anti-Ki67 (1/200; Abcam) was used, detected with the EXPOSE rabbit specific HRP/DAB detection kit (Abcam), counterstained with Hematoxylin and slides were mounted using Permount (Fisher). For immunofluorescence, anti-AREG (1/100; R&D) and anti-β-tubulin IV (1/100; BioGenex) were used and detected with appropriate secondary antibodies (1/400) conjugated to AF-555 (Thermo) and AF488 (Molecular probes). Slides were then treated against autofluorescence using 0.3% Sudan Black B (Sigma) in 70% ethanol and mounted using anti-fade medium containing DAPI (ProLong Gold from Cell Signaling Techonology). Images were taken using a Nikon Eclipse E800 (for H&E and Ki67 stains) or a Zeiss AxioImager M2 (for immunofluorescence) and analyzed using ImageJ (NIH).

### Bronchoalveolar lavage

Mice were euthanized by cervical dislocation and the lungs were lavaged twice with 0.5ml of a 0.9% saline solution. Bronchoalveolar lavage (BAL) fluid was centrifuged at 500g for 10 minutes to remove cells and debris and the supernatant was collected to quantify total protein leakage into the airway using a BCA assay (Pierce). Cell lysis and damage was analyzed from BAL fluid by measuring lactate dehydrogenase leakage using an LDH assay kit (Sigma).

### Pulmonary function phenotyping

Lung Diffusing Capacity (DF_CO_) quantifies the ability of the lung to exchange gas, which is its primary function. Diffusing capacity is simple and quick to measure in humans and mice, and it decreases with nearly all lung pathologies, including viral infections. At the selected time points, a cohort of mice was anesthetized via an IP injection of ketamine–xylazine (100 mg/kg–10 mg/kg), and then an 18-g stub needle was secured in the trachea. 0.8 mL of a gas mixture containing 0.3% neon, 0.3% CO in room air was quickly injected into the lungs, held for 9 s, then quickly withdrawn. This post breathold sample was then injected into a desktop gas chromatograph (Inficon, Micro GC model 3000A) to measure the concentrations of Ne and CO. The DF_CO_ in mice is analogous to the DL_CO_ in humans, and is calculated as 1−(CO9/COc)/(Ne_9_/Ne_c_), where subscripts c and 9 refer to the calibration gas injected and the gas from the 9 s exhaled sample. DF_CO_ is thus a dimensionless variable which varies between 0 and 1, and is used to detect the loss and recovery of lung function after the viral infections used in this study [57].

Lung mechanics: After the DF_CO_ is measured, the tracheostomy cannula was then connected to a Flexivent system (Scireq). Ventilation was accomplished at a rate of 150 breaths/minute and a tidal volume of 10 ml/kg with a PEEP of 3 cm H_2_O. A deep inspiration to 30 cmH_2_O was done, and 1 minute later the respiratory resistance (Rrs) and compliance (Crs) were measured [58]. Increased resistance reflects increased difficulty in dynamically moving air into the lung and decreased compliance reflects increased difficulty in expanding the lung parenchyma.

### Mouse tracheal epithelial cell (mTEC) cultures

For mTEC cultures, tracheas were obtained from 7–9 week old C56BL/6 female mice, digested overnight in 0.3% pronase, and enriched by depleting fibroblasts as previously described [59, 60]. The mTECs were cultured at a density of 2.22x10^5^ cells/ml on collagen-coated 24-well transwell plates for 7 days (i.e., until the cultures reached a transepithelial resistance above 1000 Ω· cm^2^) and apical medium was removed to create an air-liquid interface for 14 days to induce differentiation as described previously [60]. Cells were pre-treated for 24 h with basolateral media containing vehicle (100% ethanol) or 100nM P4 (Sigma), and injured by scratching the cell layer with a 10ul XL pipette tip, or left uninjured, and loose cells were removed by washing with media. Transepithelial cell resistance (TER) was measured prior to injury, immediately after, and every 12h for 48 h by adding 100μl of warm TEC basic media to the apical chamber. New media with vehicle or P4 was added every 24h. Every 12h, basolateral media was sampled and analyzed for AREG expression by ELISA (R&D) according to the manufacturer’s protocol. Cells were harvested in Trizol every 12h and analyzed by RT-PCR as described above.

### Statistical analyses

A power and sample size calculation was used to confirm group sizes for a power of 0.8 and contributes to differential sample sizes for some dependent measures. Repeat measures were analyzed with a multivariate analysis of variance (MANOVA) followed by planned comparisons. Discrete measures were analyzed with T-tests or two-way ANOVA followed by the Tukey method for pairwise multiple comparisons. Survival was analyzed using a Kaplan Meyer survival curve followed by a log-rank test. Mean differences were considered statistically significant if *P*<0.05.

## Supporting Information

S1 Table Cytokine and chemokine concentrations in lung homogenates from ovariectomized female mice treated with placebo (-P4) or progesterone (+P4). (DOCX)
