# Supplementary material for: Progesterone-Based Therapy Protects Against Influenza by Promoting Lung Repair and Recovery in Females
Source: PLoS Pathog. 2016 Sep 15;12(9):e1005840. doi: 10.1371/journal.ppat.1005840 (PMC5025002; doi:10.1371/journal.ppat.1005840)
Supplement: S1 Table — (DOCX) [file ppat.1005840.s001.docx]

**Supplementary table 1:** Cytokine and chemokine concentrations in lung homogenates from ovariectomized female mice treated with placebo (-P4) or progesterone (+P4).

|  |  | Days Post-Infection | | |
| --- | --- | --- | --- | --- |
| Cytokine | **Treatment** | **3** | **5** | **7** |
| IL-1β^a^ | - P4 | 928.7±135.2 | 1308.4±129.7 | 710.7±110.9 |
|  | +P4 | 1253.9±96.4 | 1576.1±227.4 | 1022.9±202.6 |
| TNF-α^a^ | - P4 | 162.7±84.4 | 235.4±16.2 | 313.0±21.0 |
|  | +P4 | 140.3±7.4 | 279.9±20.0 | 371.3±18.8* |
| IL-6^a^ | - P4 | 978.3±119.7 | 1339.2±107.4 | 700.2±92.5 |
|  | +P4 | **1207.9±2^*^** | **1478.7±177.3^*^** | **949.0±122.8^*^** |
| IFN-γ^a^ | - P4 | 82.7±6.4 | 67.7±4.9 | 3889.3±457.7^+^ |
|  | +P4 | 56.6±3.65 | 104.2±13.6 | 3092.4±399.3^+^ |
| IL-12p70^a^ | - P4 | 6.6±0.4 | 10.5±0.9^+^ | 16.4±1.2^+^ |
|  | +P4 | 5.6±0.8 | 8.4±1.5^+^ | 17.5±1.6^+^ |
| IL-4^a^ | - P4 | 37.6±10.6 | 71.6±23.6 | 52.9±26.5 |
|  | +P4 | 28.6±4.1 | 30.3±6.4 | 11.8±3.8 |
| IL-5^a^ | - P4 | 27.9±5.7 | 57.3±8.9 | 66.6±9.3 |
|  | +P4 | 42.7±13.9 | 48.6±9.9 | **110.1±10.4*** |
| IL-13^a^ | - P4 | 461.6±27.2 | 243.2±56.3 | 184.3±48.6 |
|  | +P4 | **191.6±42.0*** | 189.7±40.1 | 309.7±90.4 |
| IL-33 ^b^ | - P4 | 57.6±8.3 | 41.8±9.0 | 21.3±5.6 |
|  | +P4 | 41.0±9.9 | 28.7±5.6 | 16.9±3.4 |
| IL-10^a^ | - P4 | 25.2±4.5 | 24.2±3.1 | 158±17.9^+^ |
|  | +P4 | 26.9±4.4 | 21.1±2.7 | 178.5±25.7^+^ |
| TGF-β ^b^ | - P4 | 8299.0±1367.0 | 5953.0±469.0 | 7433.0±674.0 |
|  | +P4 | 5460.0±921.0 | 7368.0±1738.0 | **10734.0±890.0*** |

Adult female mice were ovariectomized, treated with placebo (-P4) or exogenous P4 (+P4), and inoculated with lethal IAV or mock-infected. Supernatant from whole lung homogenates was used to quantify cytokines at 3,5 and 7dpi. Data are presented as the mean ± SEM in pg/ml (a) or ng/ml (b) from two independent experiments (n=8-10/treatment/dpi). Data were analyzed with two-way ANOVA followed by Tukey tests, with significant differences compared with –P4 females at an individual time-point represented by an asterisk (*), significant differences within a treatment group at 7 or 5dpi compared with day 3 represented by a plus (+) *P*>0.05.
